# Ambulatory sleep scoring using accelerometers—distinguishing between nonwear and sleep/wake states

**DOI:** 10.7717/peerj.8284

**Published:** 2020-01-02

**Authors:** Amna Barouni, Jörg Ottenbacher, Johannes Schneider, Bernd Feige, Dieter Riemann, Anne Herlan, Driss El Hardouz, Darren McLennan

**Affiliations:** 1 movisens GmbH, Karlsruhe, Germany; 2 FZI Research Center for Information Technology, Karlsruhe Institute of Technology, Karlsruhe, Germany; 3 Department of Psychiatry and Psychotherapy, University Medical Center Freiburg, Freiburg, Germany; 4 Institute for Information Processing Technologies, Karlsruhe Institute of Technology, Karlsruhe, Germany

**Keywords:** Acceleration, Accelerometry, Sleep/Wake, Sleep scoring, Non-Wear detection, Ambulatory assessment, Nonwear

## Abstract

**Background:**

Differentiating nonwear time from sleep and wake times is essential for the estimation of sleep duration based on actigraphy data. To efficiently analyze large-scale data sets, an automatic method of identifying these three different states is required. Therefore, we developed a classification algorithm to determine nonwear, sleep and wake periods from accelerometer data. Our work aimed to (I) develop a new pattern recognition algorithm for identifying nonwear periods from actigraphy data based on the influence of respiration rate on the power spectrum of the acceleration signal and implement it in an automatic classification algorithm for nonwear/sleep/wake states; (II) address motion artifacts that occur during nonwear periods and are known to cause misclassification of these periods; (III) adjust the algorithm depending on the sensor position (wrist, chest); and (IV) validate the algorithm on both healthy individuals and patients with sleep disorders.

**Methods:**

The study involved 98 participants who wore wrist and chest acceleration sensors for one day of measurements. They spent one night in the sleep laboratory and continued to wear the sensors outside of the laboratory for the remainder of the day. The results of the classification algorithm were compared to those of the reference source: polysomnography for wake/sleep and manual annotations for nonwear/wear classification.

**Results:**

The median kappa values for the two locations were 0.83 (wrist) and 0.84 (chest). The level of agreement did not vary significantly by sleep health (good sleepers vs. subjects with sleep disorders) (*p* = 0.348, *p* = 0.118) or by sex (*p* = 0.442, *p* = 0.456). The intraclass correlation coefficients of nonwear total time between the reference and the algorithm were 0.92 and 0.97 with the outliers and 0.95 and 0.98 after the outliers were removed for the wrist and chest, respectively. There was no evidence of an association between the mean difference (and 95% limits of agreement) and the mean of the two methods for either sensor position (wrist *p* = 0.110, chest *p* = 0.164), and the mean differences (algorithm minus reference) were 5.11 [95% LoA −15.4–25.7] and 1.32 [95% LoA −9.59–12.24] min/day, respectively, after the outliers were removed.

**Discussion:**

We studied the influence of the respiration wave on the power spectrum of the acceleration signal for the differentiation of nonwear periods from sleep and wake periods. The algorithm combined both spectral analysis of the acceleration signal and rescoring. Based on the Bland-Altman analysis, the chest-worn accelerometer showed better results than the wrist-worn accelerometer.

## Introduction

Accelerometers play an important role in psycho-sociological studies, especially in the fields of sleep health (Wrzus et al., 2012; Hees et al., 2018) and sedentary behavior (Rosenberger et al., 2013). To measure sleep via actigraphy, a device that can identify nonwear periods and differentiate nonwear and sleep periods should be used (Choi et al., 2011; Winkler et al., 2012; Gorman et al., 2014). To distinguish nonwear from sleep, studies often collect supplemental data in the form of a diary (Edwardson et al., 2017). Such methods attempt to capture the sleep/wake state of the participant, the sleep quality, and the times in which the device was not worn in an effort to refine the actigraphy data. These methods place a large burden on the participant and are often inaccurate. Participants are required to provide written notes of their activity during the study and to note the start and the end of bedtime by tapping on the sensor or pressing a button. The labor requirements of the researcher for data processing remain an important consideration because such methods complicate the implementation of batch analyses (Masse et al., 2005; Edwardson et al., 2017) . Problems also arise when secondary parameters for physical activity (PA) are calculated, especially in larger data sets that require automatic wear time detection algorithms (Matthews et al., 2002). The false classification of sleep or sedentary behavior as nonwear time distorts PA estimates and results in invalid estimates of accelerometer-derived measurements of PA (King et al., 2011).

Existing algorithms classify acceleration data into wear and nonwear periods by applying a threshold to a specific metric that is calculated from the acceleration signals for each epoch (e.g., 30 s or 1 min) of a measurement. These metrics (often defined as ‘counts’) vary substantially between device manufacturers (Sadeh, Sharkey & Carskadon, 1994; Troiano et al., 2008; Kripke et al., 2010). To refine this distinction, researchers have investigated different numbers of consecutive epochs. Troiano et al. (2008) defined nonwear as a 60-minute period of consecutive minutes containing low levels of activity. Choi et al. (2011) indicated that a longer time frame of 90 min provided a better estimation of nonwear time, taking into account motion artifacts occurring during nonwear periods. Evenson & Terry (2009) observed that the propensity for low levels of physical activity to register while sleeping or sitting motionless makes sedentary and nonwear periods similar. In 2016, Kosmadopoulos, Darwent and Roach (2016) proposed an algorithm that can discern nonwear time from sleep or quiet wakefulness by comparing different periods of inactivity for a wrist-worn accelerometer (Kosmadopoulos, Darwent & Roach, 2016). In the same year, Winkler et al. (2016) presented an automated algorithm that can classify bouts of activity as nonwear and sleep periods based on knowledge of the behaviors that differentiate sleep from nonwear. The algorithm presented by Zhou et al., (2015) used both acceleration and surface skin temperature measurements to identify nonwear time. However, a temperature threshold should be calibrated for regional climates as well as for indoor/outdoor activities.

In another context, some studies have focused on extracting new features from the acceleration signal, such as the respiratory wave. With an acceleration signal that is sufficiently sensitive, a detailed analysis of the power spectrum can reveal additional features, particularly cyclical patterns such as breathing (Hung et al., 2008). Liu et al. proposed an algorithm that can determine the dynamic respiration rate during various physical activities using such an analysis. For our algorithm, we compared the existing methods of deriving the respiration rate from 3D acceleration signals, such as wavelet decomposition, non-adaptive bandpass filters and adaptive bandpass filters. Because various body motions may affect the acceleration signal, researchers combined hybrid principal component analysis (PCA) with adaptive bandpass filtering. They configured the parameters of the bandpass filter according to the energy expenditure of the individual during each physical activity (sleep, quiet sitting, sitting with minor movement, walking and running). Liu et al. investigated through their algorithm the possibility of detecting the respiration wave for different levels of activity (low, medium and high). The results confirmed that the respiration rate derived from 3D acceleration signals can withstand motion artifacts and thus can be used for ambulatory measurements (Liu et al., 2011).

After distinguishing nonwear from wear periods, the algorithm classifies remaining time into sleep and wake periods. A number of studies have focused on the usefulness of actigraphy in discriminating between sleep and wake as defined by the gold standard of polysomnography (PSG) (Mullaney, Kripke & Messin, 1980; Cole et al., 1992; Paquet, Kawinska & Carrier, 2007). The most commonly used algorithms for sleep/wake classification compare the level of activity in a defined window with a threshold or a regression algorithm based on statistical parameters derived from the acceleration signals. Sadeh, Sharkey and Carskadon proposed a regression algorithm based on the sleep/wake classification from PSG data. They used the following variables: the mean activity counts during the scored epoch and the window of five epochs preceding and following it, the standard deviation of activity counts during the scored epoch and the five epochs preceding it, the natural logarithm of the number of activity counts during the scored epoch + 1, and the number of epochs with an activity level with between 50 and 100 activity counts in a window of 11 min that includes the scored epoch and the five epochs preceding and following it (Sadeh, Sharkey & Carskadon, 1994). Cole et al. (1992) computed the weighting factors from the activity scores for a given minute, the following minutes and the previous minutes, and proposed rescoring rules to increase the accuracy rate of correct sleep/wake classification. To improve those algorithms, different decision rules have previously been proposed to differentiate bedtimes from wake times (Catrine et al., 2014) and waking wear times (McVeigh et al., 2016; Winkler et al., 2016). We focused our definitions of the rescoring rules on the common reasons behind the misclassification of sleep periods as nonwear periods (the power spectrum not being examined for respiration signals during sleep) and sleep periods as wake periods (short movements during bedtime).

To discern nonwear from sleep, existing algorithms focus on activity counts derived from the acceleration signal. The limitations of this approach include the misclassification of nonwear periods when motion artifacts are present and the inability to classify long periods of inactivity. Given the limitations of the algorithms described above, we aimed to distinguish nonwear periods from sleep periods. Therefore, we extracted a new feature from the acceleration signal and studied its ability to differentiate nonwear periods from sleep and wake periods.

## Methods

### Participants and procedure

The study included 98 participants. The healthy subjects were recruited by word of mouth. The subjects with sleep disorders (insomnia, sleep apnea, parasomnia and restless legs syndrome) were patients in the sleep medicine clinic at the University of Albert-Ludwig Freiburg Medical Center. The Ethics Committee of the University of Albert-Ludwig Freiburg granted ethical approval to carry out the study within the university facilities (Ethical Application Ref: 141/15). Written consent was obtained from the participants.

We recorded acceleration data using the activity sensor *Move3* (movisens GmbH, Karlsruhe, Germany), which was worn by the participants on both wrist and chest for one day of measurements. The accelerometers sampled the data at 64 Hz with a measurement range of ±8 g. Polysomnographic recordings were scored using the standard AASM rules (Iber et al., 2007). The participants spent one night in the sleep laboratory at the University of Albert-Ludwig Freiburg Medical Center. In the morning, an assistant recorded the nonwear periods while the participants were taking a shower. For the remainder of the day, the participants continued to wear the sensors outside of the laboratory. To identify nonwear periods occurring outside of the sleep laboratory, we referred to a change in the temperature signal from the sensor from the constant room temperature (detecting a decreasing exponential function at the beginning and an increasing exponential function at the end of a nonwear period) and a visual evaluation of acceleration signal (Boyne et al., 2013). The PSG results served as a reference for the sleep and wake periods.

In this work, we defined an “epoch” as a basic time segment with a fixed length of 30 s, a “window” as a time segment composed of a fixed number of consecutive epochs (e.g., 10 min or 20 min), and a “period” as an undefined number of consecutive epochs (e.g., a period of sleep, a period of wake).

### Statistical analysis

The statistical analyses were performed in R (Version 3.4.1). The normality of the data was tested for each study group separately using the Anderson-Darling normality test. To describe the demographic data, the categorical variables were summarized as counts, and the continuous variables with nonnormal distributions were expressed as medians and IQRs. To compare the two study groups (healthy subjects vs. patients with sleep disorders), the chi-square test was used for nominal data, and the Mann–Whitney *U* test was used for continuous data with a nonnormal distribution.

The goal of the study was to investigate the agreement between the actigraphy-based algorithm and nonwear annotations. Therefore, we evaluated the kappa values for each participant (reported as median and 25th and 75th percentiles because these data were skewed): agreement was “poor” if kappa <0, “slight” if 0 ≤kappa ≤0.20, “fair” if 0.21 ≤kappa ≤0.40, “moderate” if 0.41 ≤kappa ≤0.60, “substantial” if 0.61 ≤kappa ≤0.80, and “almost perfect” if kappa>0.80 (Landis & Koch, 1977). We then compared the kappa values across different groups (sex, healthy subjects/patients with sleep disorders) using the Mann–Whitney-U test with a significance level of *p* < 0.05. Furthermore, we compared the total nonwear time from the actigraphy-based algorithm versus the annotation using the Bland-Altman approach (Bland & Altman, 1986) to calculate the mean of the difference and the 95% limits of agreement. We also presented the linear regression results of the difference in the mean and 95% limits of agreement. Finally, we determined the intraclass correlation coefficient (ICC) (model “two-way”, type “agreement”, effect “random”) to assess the interrater reliability (Koo & Li, 2016) and the Pearson correlation coefficient to evaluate the linear relationships of total nonwear time between the algorithm and the reference.

For the sleep/wake analysis, as the PSG is the gold standard, we evaluated sensitivity, specificity, the positive predictive value, the negative predictive value, informedness (two-class analysis), accuracy, and the kappa value. We also performed an epoch-by-epoch (30 s) analysis by computing the confusion matrix (Kohavi & Provost, 1998).

### Algorithm

Structuring the proposed algorithm required precise observations of sleep and nonwear periods and considered the main differences between them. The composition of the algorithm consisted of the following steps:

1. Collect raw 3D accelerometer data at a sampling rate of 64 Hz and resolution of 4 mg.
2. Preprocess the signal by using bandpass filters (Butterworth filter: frequency = 0.25–3 Hz, order = 4), threshold-crossing (threshold = 0.015 over 30 s).
3. Apply the nonwear detection algorithm.
4. Apply the sleep detection algorithms.
5. Apply the rescoring rules to correct misclassifications.

We used five sets of measurements that were acquired during the study but were not included in the evaluation to graphically determine the thresholds by trial and error. The participants wore the same devices and followed the same protocol as described in the study. These measurements were chosen randomly.

### Nonwear algorithm

We evaluated the power spectrum of the three-dimensional acceleration signal to identify nonwear epochs. The power spectrum for wake and sleep periods showed a high amplitude in the 0.1 Hz–0.4 Hz frequency range (representing the range of respiration frequencies), and the power spectrum of the nonwear periods showed a low intensity across the total frequency range. We used this observation to discern nonwear epochs by identifying a reduced power spectrum in the 0.1 Hz to 0.4 Hz range. The power within this frequency band was computed for each signal (*x*, *y*, *z*) over a 10-minute window. Afterwards, the maximum power *Pmax* was determined as the maximum power over the three axes. We classified the event as nonwear if the maximum power *Pmax* fell below the threshold *P0* = 2*10^−5^:

\begin{eqnarray*}& & \text{If} Pmax\geq P0,\text{then the epoch is classified as a wear epoch} \end{eqnarray*}

\begin{eqnarray*}& & \text{If} Pmax\lt P0,\text{then the epoch is classified as a nonwear epoch} \end{eqnarray*}

### Sleep algorithm

After applying a 0.25–3 Hz bandpass filter to the accelerometer signal, a specific metric (we used threshold-crossing) was calculated over each 30-second epoch. Within each sliding window of 20 min, the number of epochs with zero metrics were summed and compared with the threshold *Th0* = 15. If the resulting value was above *Th0*, the window was classified as a sleep window. For the longest sleep period in the day (from noon to noon), sleep onset was defined as the first ten consecutive sleep epochs.

### Rescoring rules

**Figure 1:**
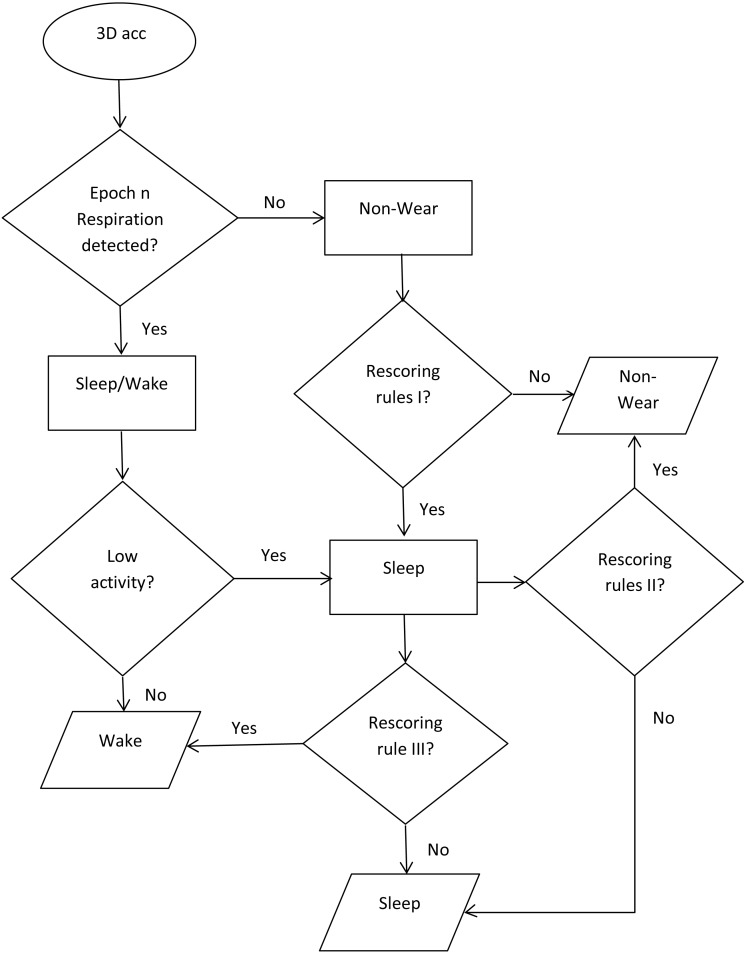
Flowchart of the classification algorithm. Classification of the three states: nonwear, sleep, wake.

To develop the automated sleep time and nonwear time detection algorithm, we used the calculations for nonwear and sleep time and then implemented the following rescoring criteria (Fig. 1):

1. Incorrectly classified nonwear epochs occurring during sleep were rescored. First, we identified the nonwear period (surrounded by sleep periods). Then, we analyzed the power spectrum of the period, and if 75% of the nonwear period indicated respiration, we reclassified the period as a sleep period. This verification step prevented the misclassification of a real nonwear period during sleep, where the subject removed the sensor for a short period and replaced it.
2. Misclassifications of a nonwear period occurring at bedtime or just before bedtime (i.e., the participant removing the sensor just before or after entering the bed) were corrected. After identifying the period (presenting unusually high activity from one side), the whether the power spectrum indicated respiration for over 75% of the period was assessed.
3. Movements lasting longer than one minute during sleep were considered as movements occurring during wake.

## Results

The study involved 98 participants: 55 males and 43 females with a median age of 28.5 years (range 19–63 years, IQR 15.7 years). We split the data into three subsamples for the analyses. The first sample contained all subjects (98), the second only healthy subjects (48), and the third consisted of patients with sleep disorders (50). We excluded 5 subjects due to a lack of annotation of the sleep periods.

The median measurement duration was 952.7 min (IQR 89.2 min) per day, with a median estimated total nonwear time of 49.7 min/day (IQR 49.3 min) and a median total sleep time of 412.2 min/day (IQR 49.1 min) (Table 1). For the epoch-by-epoch analysis, we used an epoch length of 30 s and compared the concordance of the actigraphy-based classification algorithm with the PSG data and nonwear annotations. The rows of the confusion matrix present the wake, sleep and nonwear classes as determined by the PSG data and nonwear annotations, and the columns contain the classes of the epochs as determined by the actigraphy data. Among all the nonwear epochs, 90.1% and 93.5% were identified as nonwear, 2.0% and 0.0% as sleep, and 7.8% and 6.4% as wake for the wrist and chest, respectively. Of all the sleep epochs, 92.2% and 97.1% were classified as sleep, 0.2% and 0.02% were classified as nonwear, and 7.5% and 2.8% were classified as wake for the wrist and chest, respectively. Moreover, among all of the wake epochs, 87.9% and 85.4% were classified as wake, 10.8% and 13.8% as sleep, and 1.2% and 0.6% as nonwear for the wrist and chest, respectively (Table 2, Table 3).

**Table 1:** Demographic data of participants.

Characteristics	Overall (*n* = 98)	Healthy (*n* = 48)	Patient with sleep disorders (*n* = 50)	p^*^
Sex (Male/Female)	43/55	23/25	20/30	0.558^a^
Age (years)	28.5(15.7)	25.5(9.2)	33.5(18.2)	0.006^b^
Total sleep time (min) from PSG	412.2(49.1)	419.7(44.7)	410(56.6)	0.682^b^
Total nonwear time (min) from manual reference	49.7(49.3)	32.7(15.7)	78.5(50.0)	<0.001^b^
Length of measurement (min) from accelerometer sensor	952.7(89.2)	959.5(36.0)	949(208.2)	0.009^b^

**Notes.**

Median (IQR).

IQR, interquartile range.

a Chi-square test.

b Mann–Whitney-*U* test.

* *p* for difference between participants with and without sleep disorders (*p* < 0.05 considered statistically significant).

**Table 2:** Confusion matrix for wrist-worn activity sensor.

	**Reference: number of epochs (30-sec) (% of column)**			
**Algorithm**	**Wake wear**	**Sleep wear**	**Nonwear**	**Total**
Wake wear	74,735 (87.91%)	5,562 (7.50%)	855 (7.83%)	81,152
Sleep wear	9,219 (10.84%)	68,372 (92.26%)	222 (2.03%)	77,813
Nonwear	1,054 (1.23%)	168 (0.22%)	9,841 (90.13%)	11,063
Total	85,008	74,102	10,918	170,028

**Table 3:** Confusion matrix for chest-worn activity sensor.

	**Reference: number of epochs (30-sec) (% of column)**			
**Algorithm**	**Wake wear**	**Sleep wear**	**Nonwear**	**Total**
Wake wear	73,077 (85.47%)	2,094 (2.82%)	676 (6.48%)	75,847
Sleep wear	11,856 (13.86%)	71,992 (97.15%)	0 (0%)	83,848
Nonwear	567 (0.66%)	16 (0.02%)	9,747 (93.51%)	10,330
Total	85,500	74,102	10,423	170,025

The algorithm achieved a median sensitivity of 0.92 and 0.95 for the wrist and chest, respectively, and a specificity of 1, 0.99 for nonwear; a median sensitivity of 0.90 and 0.87, respectively, and specificity of 0.94, 0.98 for sleep; and a median sensitivity of 0.93 and 0.97, respectively, and specificity of 0.90, 0.88 for wake (Tables 4 and 5). The median accuracy was 0.90 and 0.91 for the wrist and chest, respectively, and the median kappa values were 0.83 and 0.84, respectively. “Almost perfect agreement” occurred for 63.4% and 67.7% of the participants, 33.3% and 27.9% of the participants had “substantial agreement”, and 3.2% and 4.3% of the participants had “moderate agreement” for the wrist and chest, respectively. There were no occurrences of “poor agreement” and “fair agreement” (Landis & Koch, 1977) . We compared the performance of the classification algorithms across several participant characteristics. Agreement did not vary significantly by sex (*p* = 0.215 and *p* = 0.456 for the wrist and chest, respectively) or by study group between the healthy subjects and patients with sleep disorders (*p* = 0.348 and *p* = 0.118) (Table 6).

**Table 4:** Agreement between the algorithm and the reference: wrist.

	**Automated algorithm wrist nonwear** **median [25**th**;75**th **percentile]**	**Automated algorithm wrist sleep** **median [25**th**;75**th **percentile]**	**Automated algorithm wrist wake** **median [25**th**;75**th **percentile]**
N	93	93	93
Sensitivity	0.92[0.85;0.94]	0.90[0.83;0.93]	0.93[0.88;0.98]
Specificity	1.00[0.99;1.00]	0.94[0.90;0.98]	0.90[0.85;0.93]
PPV	1.00[0.96;1.00]	0.95[0.87;0.98]	0.89[0.84;0.93]
NPV	0.99[0.98;0.99]	0.91[0.87;0.94]	0.94[0.88;0.98]
Informedness	0.92[0.85;0.94]	0.83[0.75;0.89]	0.82[0.73;0.87]

**Table 5:** Agreement between the algorithm and the reference: chest.

	**Automated algorithm chest nonwear** **median [25**th**;75**th **percentile]**	**Automated algorithm chest sleep** **median [25**th**;75**th **percentile]**	**Automated algorithm chest wake** **median [25**th**;75**th **percentile]**
N	93	93	93
Sensitivity	0.95[0.92;0.97]	0.87[0.81;0.93]	0.97[0.94;0.98]
Specificity	0.99[0.99;1.00]	0.98[0.96;0.99]	0.88[0.82;0.93]
PPV	0.97[0.91;1.00]	0.98[0.96;0.99]	0.86[0.80;0.91]
NPV	0.99[0.98;0.99]	0.89[0.82;0.93]	0.97[0.96;0.98]
Informedness	0.95[0.92;0.97]	0.85[0.78;0.89]	0.84[0.78;0.89]

**Table 6:** Effect of sex and study group on agreement between the algorithm and the reference for wrist and chest.

**Statistic**	**Automated algorithm wrist** **median [25th, 75th percentile]**	**Automated algorithm chest** **median [25th, 75th percentile]**
Accuracy (overall)	0.90[0.87;0.93]	0.91[0.88;0.94]
Kappa (overall)	0.83[0.75;0.88]	0.84[0.78;0.89]
Slight/ Poor agreement (k <=0.2), *n*(%) (Landis & Koch, 1977)	0(0%)	0(0%)
Fair agreement (0.2 < k <= 0.4), *n*(%)	0(0%)	0(0%)
Moderate agreement (0.4 < k <= 0.6), *n*(%)	3(3.23%)	4(4.30%)
Substantial agreement (0.6 < k <= 0.8), *n*(%)	31(33.33%)	26(27.96%)
Almost perfect agreement (0.8 < k), *n*(%)	59(63.44%)	63(67.74%)
Kappa, men	0.81[0.75;0.87]	0.83[0.78;0.89]
Kappa, women	0.84[0.78;0.88]	0.85[0.78;0.89]
P^a^ for difference sex	0.215	0.456
Kappa, healthy	0.83[0.76;0.88]	0.85[0.80;0.90]
Kappa, patient	0.81[0.74;0.87]	0.82[0.76;0.88]
P^a^ for difference study group	0.348	0.118

**Notes.**

a Mann–Whitney-*U* test.

**Figure 2:**
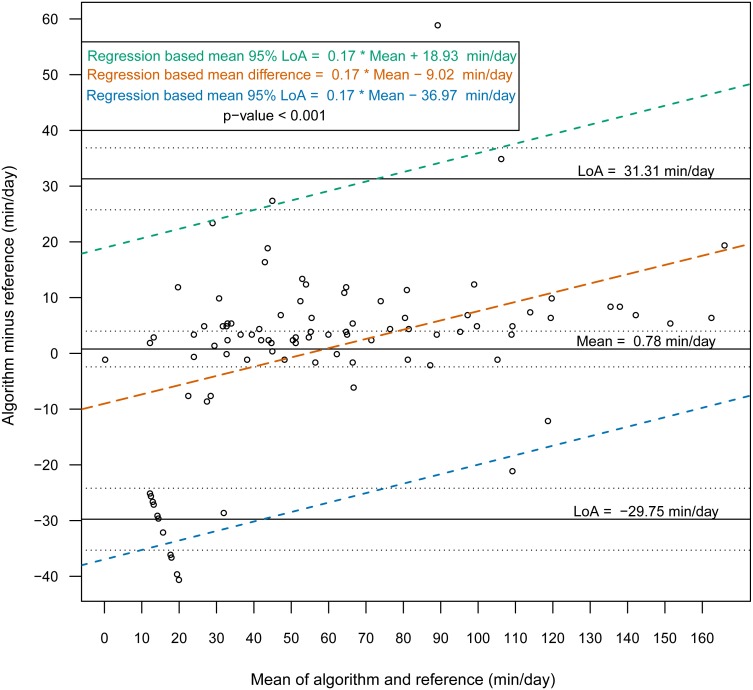
Bland-Altman plot of individual differences between algorithm and reference for total nonwear time: wrist (before removing the outliers). Bland-Altman plots with dotted lines indicating the 95% limits of agreement (LoA) and straight line indicating the mean. Dashed lines represent the regression functions of mean of difference (tenne), upper 95% LoA (dark cyan–lime green), and lower 95% LoA (cerulean) (*n* = 93).

**Figure 3:**
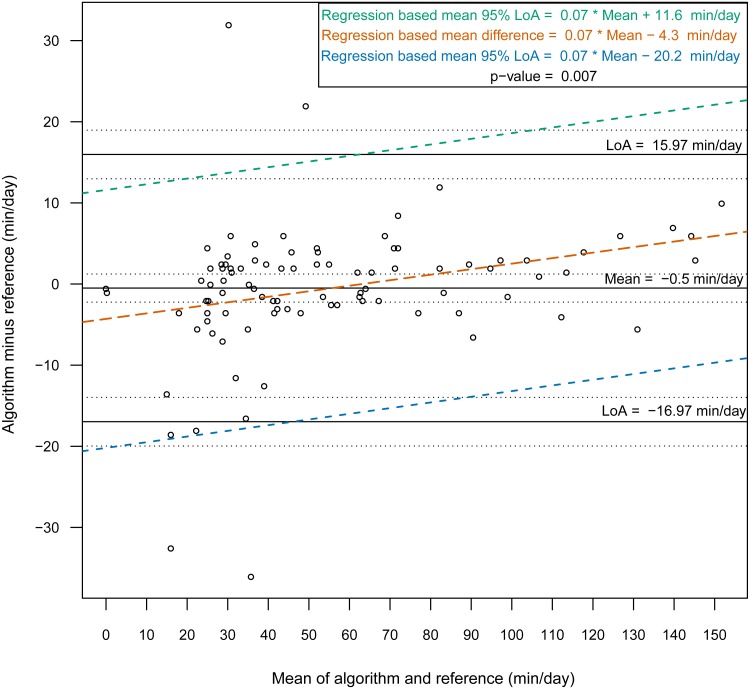
Bland-Altman plot of individual differences between algorithm and reference for total nonwear time: chest (before removing the outliers). Bland-Altman plots with dotted lines indicating the 95% limits of agreement (LoA) and straight line indicating the mean. Dashed lines represent the regression functions of mean of difference (tenne), upper 95% LoA (dark cyan–lime green), and lower 95% LoA (cerulean) (*n* = 93).

**Figure 4:**
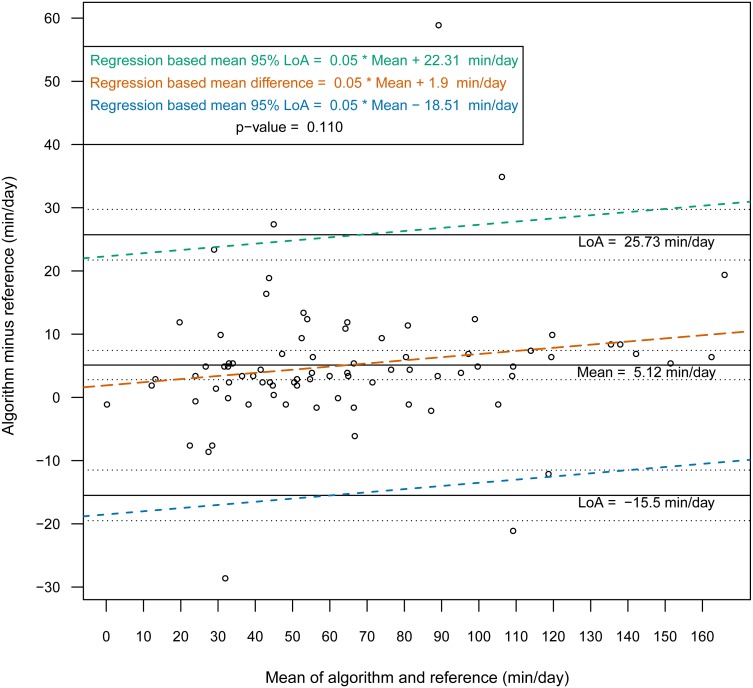
Bland-Altman plot of individual differences between actigraphy and reference for total nonwear time: wrist (after removing the outliers). Bland-Altman plots with dotted lines indicating the 95% limits of agreement (LoA) and straight line indicating the mean. Dashed lines represent the regression functions of mean of difference (tenne), upper 95% LoA (dark cyan–lime green), and lower 95% LoA (cerulean) (*n* = 81).

**Figure 5:**
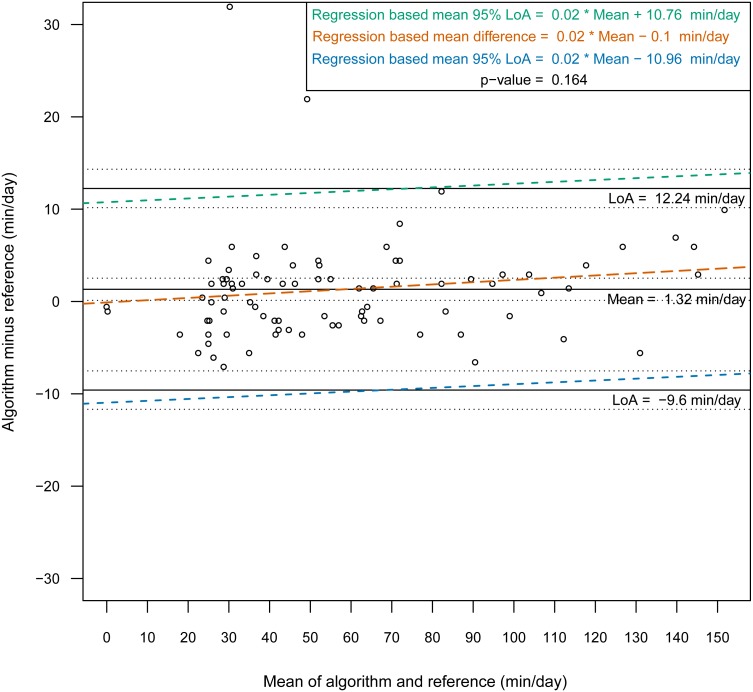
Bland-Altman plot of individual differences between actigraphy and reference for total nonwear time: chest (after removing the outliers). Bland-Altman plots with dotted lines indicating the 95% limits of agreement (LoA) and straight line indicating the mean. Dashed lines represent the regression functions of mean of difference (tenne), upper 95% LoA (dark cyan–lime green), and lower 95% LoA (cerulean) (*n* = 85).

Then, we constructed Bland-Altman plots for each sensor position to assess the differences in nonwear time per day between the algorithm and the reference (Figs. 2 and 3). For wrist- and chest-worn accelerometers, the mean differences (algorithm minus reference) were 0.7 [95% LoA −29.7–31.3] and −0.5 [95% LoA −16.9–15.9] min/day, respectively. The linear regression results of the difference in the mean and the 95% LoA showed an upward trend for higher measurements. The intraclass correlation coefficient between the algorithm and the reference was 0.92 (95% CI [0.88–0.94], *p* < 0.001) for the wrist and 0.97 (95% CI [0.95–0.98], *p* < 0.001) for the chest (Table 7). We also investigated the Bland-Altman plots after removing 12 and 8 outlier measurements for the wrist and chest, respectively, that had inaccurate counts of nonwear epochs (Figs. 4 and 5). These inaccuracies occurred due to misclassifications of short nonwear periods that were directly adjacent to sleep periods and were thus considered as sleep periods and misclassifications of nonwear periods with unusual high levels of activity that were thus classified as wake periods. For the wrist- and chest-worn accelerometers, the slopes of the linear regressions of the difference in the mean and the 95% LoA were 0.05 (*p* = 0.110) and 0.02 (*p* = 0.164), respectively, and the mean differences (algorithm minus reference) were 5.11 [95% LoA −15.4–25.7] and 1.32 [95% LoA −9.59–12.24] min/day, respectively. The ICC between the algorithm and the reference after the outliers were removed was 0.95 (95% CI [0.90–0.97], *p* < 0.001) for wrist and 0.98 (95% CI [0.97–0.99], *p* < 0.001) for chest (Table 8).

## Discussion

This study evaluated a newly developed automated algorithm for nonwear/sleep/wake classification based on acceleration data recorded from different locations on the body (wrist and chest). A frequency spectrum-based algorithm (Liu et al., 2011), which combined hybrid PCA and adaptive bandpass filtering, was designed to estimate the respiratory rate according to body activities (sleep, quiet sitting, sitting with minor movements, walking and running) within the range 0.2–0.7 Hz. In our work, we implemented the same principle to discern nonwear epochs from wear epochs by studying the power spectrum of the acceleration signal and determining an estimation of the respiration rate. We found that a power spectral analysis within the range 0.1–0.4 Hz was sufficient for nonwear/wear classification, as the spectrum presented a low power in this range when the sensor was not worn. However, we noted from the confusion matrix of the epoch-by-epoch classification of the three states nonwear/sleep/wake (Tables 2 and 3) that nonwear was more often wrongly classified as wake (7.8%, 6.4%) than as sleep (2.0%, 0.0%). The misclassification of nonwear as wake could also occur via the accumulation of long movements during nonwear periods, with participants relocating the sensor, especially at the beginning and end of a nonwear period, resulting in the appearance of a high power in the range 0.1–0.4 Hz. The analysis of total nonwear time indicated that the upper 95% LoA is half as large for chest-worn sensors versus wrist-worn sensors. As other studies have noted that wrist monitors are less biased than waist monitors (Choi et al., 2012), the algorithm based on the spectral analysis of the respiration wave may be more appropriate for chest-worn sensors. The 95% LoA was shorter than 32 min for both the wrist- and the chest-worn sensors. This result means that wear periods were classified as nonwear periods due to the absence of the respiration wave by the spectral analysis. Those periods were mostly present during sleep when the sensor was removed. As different studies have suggested the use of 90 min (Choi et al., 2012) or 60 min (Keadle et al., 2014) of consecutive zeros as the criterion, we can conclude that the bias had no practical significance. According to the comparison of the Bland-Altman plots before (Figs. 2 and 3) and after the removal of the outliers (Figs. 4 and 5), the bias was not constant over the range of values before the outliers were removed. When short nonwear periods were directly adjacent to sleep periods, the algorithm considered them to be sleep periods due to the lack of large changes in activity between the two periods. In fact, the investigated period included both sleep and nonwear. Therefore, after the second rescoring rule was applied, the respiration signal derived from the power spectrum covered more than 75% of the period and thus enabled the classification of the period as sleep. This result led to the conclusion that the second rule should be revised either by adjusting the percentage value from 75% or by defining a new method to adjust the limits of the period suspected to be a nonwear period.

**Table 7:** Bland–Altman statistic parameters for total nonwear time for wrist and chest (before removing the outliers).

**Characteristic**	**Algorithm** **mean(sd)**	**Reference** **mean(sd)**	**Mean of the difference**^c^	**95% limits of agreement**	**Intraclass correlation coefficient** **ICC**	**Pearson correlation**
Nonwear min/day **(wrist) (*n* = 93)**	59.47(42.65)	58.69(36.31)	0.77 [−2.42;3.98]^a^ *p*-value=0.630^b^	−31.30;29.75	0.92 [0.88;0.94]^a^ *p*-value <0.001	0.93 [0.90;0.95]^a^ *p*-value <0.001
Nonwear min/day **(chest) (*n* = 93)**	55.53(35.99)	56.03(33.65)	−0.5 [−2.23;1.23]^a^ *p*-value = 0.567^b^	−16.96;15.96	0.97 [0.95;0.98]^a^ *p*-value <0.001	0.97 [0.95;0.98]^a^ *p*-value <0.001

**Notes.**

a 95% Confidence interval.

b One sample *t*-test.

c algorithm minus reference.

Annotating the nonwear periods using only a diary as a reference required the verification of compliance and whether the time-stamps in the diary matched those derived from the acceleration signal (Masse et al., 2005) . To improve the quality of nonwear annotations, we referred to visual assessments of the acceleration signal (to find long continuous bouts of inactivity) (McVeigh et al., 2016) and a visual examination of the temperature signal, which showed a decreasing exponential function at the beginning of the nonwear period and an increasing exponential function immediately after the activity sensor was donned. This finding introduces the question of why researchers do not use temperature instead of acceleration to identify nonwear from wear periods or combine both methods. Zhou et al. presented the performance of classification algorithms based on acceleration and temperature data, first separately and then combined. While the classification algorithm based on temperature showed a higher value for sensitivity (0.93) than for specificity (0.64), the classification algorithm based on acceleration had a sensitivity of 0.76 and a specificity of 0.89. By combining both methods, a sensitivity of 0.94 and a specificity of 0.91 were achieved (Zhou et al., 2015). However, the variation in temperature from situations such as indoor or outdoor activities can impact the performance of the combined classification algorithm. The performance depended on the temperature, which makes it difficult to use the algorithm in different climate conditions. On the other hand, our classification algorithm, based only on acceleration data, achieved similar sensitivity and specificity as the classification algorithm based on a combination of acceleration and temperature data.

After defining nonwear epochs, we discerned sleep from wake based on the level of activity within a defined timeframe using a simple threshold-based algorithm. The thresholds of the algorithm were determined using PSG, which is the gold-standard measure of sleep parameters for laboratory research and is the best method for validating other classification methods, such as activity sensors. At the end of the algorithm, we proposed rescoring rules based on visual assessments to correct the misclassifications accrued by traditional classification algorithms due to issues such as motion artifacts that occur during nonwear periods (e.g., when the sensor is nudged or touched while being placed on a table or nightstand) and long motionless periods with sporadic movements during sleep.

Nonwear/sleep/wake classification studies differ in how the reference is collected. In (McVeigh et al., 2016), in-bed periods (the period from when the participants went to bed to the time at which they arose from the bed) were identified by two independent raters, and these periods included the time the participant spent trying to fall asleep, the time he or she was asleep, and the duration of wakefulness after sleep onset. When PSG is used as a reference, sleep time can be accurately identified, as PSG reports in-bed time as well as sleep time. However, PSG remains restricted to sleep laboratories, and sleep periods during the day (e.g., napping) cannot be classified. Thus, visual identification or diaries remains essential for determining the reference data for sleep/wake analyses from data in real world environments outside of the lab. We also found that 10.8% (for the wrist) and 13.9% (for the chest) of the wake epochs, e.g., epochs occurring during long motionless periods of wakefulness (insomnia) or between the beginning of an in-bed time period and sleep onset, were misclassified as sleep epochs. Nevertheless, an additional feature is needed to accurately determine sleep onset times, even though we defined sleep onset as the beginning of ten consecutive sleep epochs.

**Table 8:** Bland-Altman statistic parameters for total nonwear time for wrist and chest (after removing the outliers).

**Characteristic**	**Algorithm** **mean(sd)**	**Reference** **mean(sd)**	Mean of the difference^c^	95% limits of agreement	Intraclass correlation coefficient **ICC**	Pearson correlation
Nonwear min/day **(wrist) (*n* = 81)**	67.45 (39.00)	62.34 (37.15)	5.11 [2.80;7.42]^a^ *p*-value <0.001^b^	−15.49;25.73	0.95 [0.90;0.97]^a^ *p*-value <0.001	0.96 [0.94;0.97]^a^ *p*-value <0.001
Nonwear min/day **(chest) (*n* = 85)**	59.20 (35.35)	57.87 (34.51)	1.32 [0.12;2.52]^a^ *p*-value=0.031^b^	−9.59;12.24	0.98 [0.97;0.99]^a^ *p*-value <0.001	0.98 [0.98;0.99]^a^ *p*-value <0.001

**Notes.**

a 95% Confidence interval.

b One sample *t*-test.

c algorithm minus reference.

## Limitations of the Study

The wrist- and chest-worn accelerometer data were collected in a sleep laboratory, which can influence the behavior of the participants. We had no additional information about sleep periods occurring during the day, as the sleep reference data was defined based only on PSG measurements, which is the gold-standard method of assessing sleep. The rest of the day was thus considered as wake time. The annotations of the nonwear reference was performed manually based on knowledge of the study procedure (report of the assistant in the sleep laboratory), the visual assessments of acceleration signal and temperature signal and were therefore inaccurate. However, the combination of both methods allowed more accurate nonwear references. The thresholds presented in the algorithm were determined from data that was collected as part of the study from different participants but was not used in the analyses. The acceleration signal was collected from one sensor brand, which can influence the choice of the thresholds and the results when using the algorithm with other sensors presenting different noise levels.

## Conclusion

Using accelerometers yields new insights and opportunities in a wide range of psycho-sociological studies, especially in the field of sleep health. We propose an algorithm that can differentiate nonwear periods from sleep and wake periods based on the detection of the respiration wave. Future studies should focus on sleep and wake classification using other complementary methods besides threshold-based methods, such as frequency analysis of the acceleration signal and differences in respiration patterns, during sleep and wake states as well as during sedentary behaviors, as this information is especially valuable when short nap periods are of interest in the context of ambulatory studies. These methods could also be used to determine the exact start and end times of sleep using acceleration data.

##  Supplemental Information

10.7717/peerj.8284/supp-1 Dataset S1 Reference data for on-wrist worn accelerometer 0: wake, 1: sleep, 2: non-wear.

10.7717/peerj.8284/supp-2 Dataset S2 Reference data for on-chest worn accelerometer 0: wake, 1: sleep, 2: non-wear.

10.7717/peerj.8284/supp-3 Dataset S3 Algorithm data for on-wrist worn accelerometer 0: wake, 1: sleep, 2: non-wear.

10.7717/peerj.8284/supp-4 Dataset S4 Algorithm data for on-chest worn accelerometer 0: wake, 1: sleep, 2: non-wear.

10.7717/peerj.8284/supp-5 Dataset S5 Demographic data of participants (chest and wrist) Study Group (healthy, patient), Sex (M, male, F, female), Age.

## Additional Information and Declarations

### Data Availability

The following information was supplied regarding data availability: The raw data collected from the reference and the classification algorithm are available in the Supplementary Files.

## References

[ref-1] Bland JM, Altman DG (1986). Statistical methods for assessing agreement between two methods of clinical measurement. The Lancet.

[ref-2] Boyne K, Sherry DD, Gallagher PR, Olsen M, Brooks LJ (2013). Accuracy of computer algorithms and the human eye in scoring actigraphy. Sleep & Breathing = Schlaf & Atmung.

[ref-3] Catrine T-L, Barreira Tiago V, Schuna Jr JM, Mire Emily F, Katzmarzyk Peter T (2014). Fully automated waist-worn accelerometer algorithm for detecting children’s sleep-period time separate from 24-h physical activity or sedentary behaviors. Applied Physiology, Nutrition, and Metabolism.

[ref-4] Choi L, Liu Z, Matthews CE, Buchowski MS (2011). Validation of accelerometer wear and nonwear time classification algorithm. Medicine and Science in Sports and Exercise.

[ref-5] Choi L, Ward SC, Schnelle JF, Buchowski MS (2012). Assessment of wear/nonwear time classification algorithms for triaxial accelerometer. Medicine and Science in Sports and Exercise.

[ref-6] Cole RJ, Kripke DF, Gruen W, Mullaney DJ, Gillin JC (1992). Automatic sleep/wake identification from wrist activity. Sleep.

[ref-7] Edwardson CL, Winkler EAH, Bodicoat DH, Yates T, Davies MJ, Dunstan DW, Healy GN (2017). Considerations when using the activPAL monitor in field-based research with adult populations. Journal of Sport and Health Science.

[ref-8] Evenson KR, Terry JWJ (2009). Assessment of differing definitions of accelerometer nonwear time. Research Quarterly for Exercise and Sport.

[ref-9] Gorman E, Hanson HM, Yang PH, Khan KM, Liu-Ambrose T, Ashe MC (2014). Accelerometry analysis of physical activity and sedentary behavior in older adults: a systematic review and data analysis. European Review of Aging and Physical Activity.

[ref-10] Hees V, Theodoor V, Sabia S, Jones SE, Wood AR, Anderson KN, Kivimäki M, Frayling TM, Pack AI, Bucan M, Trenell MI, Mazzotti DR, Gehrman PR, Singh-Manoux BA, Weedon MN (2018). Estimating sleep parameters using an accelerometer without sleep diary. Scientific Reports.

[ref-11] Hung PD, Bonnet S, Guillemaud R, Castelli E, Yen PTN (2008). Estimation of respiratory waveform using an accelerometer.

[ref-12] Iber C, Ancoli-Israel S, Chesson A, Quan SF (2007). The AASM manual for the scoring of sleep and associated events: rules, terminology and technical specifications.

[ref-13] Keadle SK, Shiroma EJ, Freedson PS, Lee I-M (2014). Impact of accelerometer data processing decisions on the sample size, wear time and physical activity level of a large cohort study. BMC Public Health.

[ref-14] King WC, Li J, Leishear K, Mitchell JE, Belle SH (2011). Determining activity monitor wear time: an influential decision rule. Journal of Physical Activity and Health.

[ref-15] Kohavi R, Provost F (1998). Glossary of terms. Machine Learning.

[ref-16] Koo TK, Li MY (2016). A guideline of selecting and reporting intraclass correlation coefficients for reliability research. Journal of Chiropractic Medicine.

[ref-17] Kosmadopoulos A, Darwent D, Roach GD (2016). Is it on? An algorithm for discerning wrist-accelerometer non-wear times from sleep/wake activity. Chronobiology International.

[ref-18] Kripke DF, Hahn EK, Grizas AP, Wadiak KH, Loving RT, Poceta JS, Shadan FF, Cronin JW, Kline LE (2010). Wrist actigraphic scoring for sleep laboratory patients: algorithm development. Journal of Sleep Research.

[ref-19] Landis JR, Koch GG (1977). The measurement of observer agreement for categorical data. Biometrics.

[ref-20] Liu G-Z, Guo Y-W, Zhu Q-S, Huang B-Y, Wang L (2011). Estimation of respiration rate from three-dimensional acceleration data based on body sensor network. Telemedicine Journal and E-Health.

[ref-21] Masse LC, Fuemmeler BF, Anderson CB, Matthews CE, Trost SG, Catellier DJ, Treuth M (2005). Accelerometer data reduction: a comparison of four reduction algorithms on select outcome variables. Medicine and Science in Sports and Exercise.

[ref-22] Matthews CE, Ainsworth BE, Thompson RW, Bassett DR (2002). Sources of variance in daily physical activity levels as measured by an accelerometer. Medicine and Science in Sports and Exercise.

[ref-23] McVeigh JA, Winkler EAH, Healy GN, Slater J, Eastwood PR, Straker LM (2016). Validity of an automated algorithm to identify waking and in-bed wear time in hip-worn accelerometer data collected with a 24 h wear protocol in young adults. Physiological Measurement.

[ref-24] Mullaney DJ, Kripke DF, Messin S (1980). Wrist-actigraphic estimation of sleep time. Sleep.

[ref-25] Paquet J, Kawinska A, Carrier J (2007). Wake detection capacity of actigraphy during sleep. Sleep.

[ref-26] Rosenberger ME, Haskell WL, Albinali F, Mota S, Nawyn J, Intille S (2013). Estimating activity and sedentary behavior from an accelerometer on the hip or wrist. Medicine and Science in Sports and Exercise.

[ref-27] Sadeh A, Sharkey M, Carskadon MA (1994). Activity-based sleep-wake identification: an empirical test of methodological issues. Sleep.

[ref-28] Troiano RP, Berrigan D, Dodd KW, Masse LC, Tilert T, McDowell M (2008). Physical activity in the United States measured by accelerometer. Medicine and Science in Sports and Exercise.

[ref-29] Winkler EAH, Bodicoat DH, Healy GN, Bakrania K, Yates T, Owen N, Dunstan DW, Edwardson CL (2016). Identifying adults’ valid waking wear time by automated estimation in activPAL data collected with a 24 h wear protocol. Physiological Measurement.

[ref-30] Winkler EAH, Gardiner PA, Clark BK, Matthews CE, Owen N, Healy GN (2012). Identifying sedentary time using automated estimates of accelerometer wear time. British Journal of Sports Medicine.

[ref-31] Wrzus C, Brandmaier AM, Von Oertzen T, Muller V, Wagner GG, Riediger M (2012). A new approach for assessing sleep duration and postures from ambulatory accelerometry. PLOS ONE.

[ref-32] Zhou S-M, Hill RA, Morgan K, Stratton G, Gravenor MB, Bijlsma G, Brophy S (2015). Classification of accelerometer wear and non-wear events in seconds for monitoring free-living physical activity. BMJ Open.

